# Social Media Effects on Public Trust in the European Union

**DOI:** 10.1093/poq/nfad029

**Published:** 2023-08-03

**Authors:** Osman Sabri Kiratli

**Affiliations:** Associate Professor, International Trade Department, Bogazici University, Istanbul, Turkey; and Visiting Research Fellow, Global Governance Unit, WZB Berlin, Berlin, Germany

## Abstract

This paper scrutinizes the effect of social media use on institutional trust in the European Union (EU) among European citizens. Fixed-effects regression models on data from the Eurobarometer survey conducted in 2019, the year of the most recent European Parliament (EP) elections, demonstrate that higher social media use is associated with lower trust in the EU. More importantly, social media usage habits exert particularly detrimental effects in regions with wider and faster internet connections. In such high-information environments, those who more frequently use online social networks, tend to trust those networks, and receive information on EU affairs from these networks have less faith in the EU compared to those in regions with lower-quality internet access. In contrast, in regions with lower broadband access, receiving EU information from social media fosters political trust.

## Social Media Effects on Public Trust in the European Union

The political consequences of the expansion of high-speed internet and social media are well documented (see Lorenz-Spreen et al. 2023). Studies show that growing internet penetration results in lower voter turnouts (Falck, Gold, and Heblich 2014; Gavazza, Nardotto, and Valletti 2019; Guriev, Melnikov, and Zhuravskaya 2021), contributes to electoral uncertainty (Sudulich, Wall, and Baccini 2015), and harms the electoral prospects of ruling parties (Zimmermann and Kohring 2020). In European countries that experienced a rapid expansion of broadband availability and 3G networks, populist parties reportedly saw their vote shares rise (e.g., Campante, Durante, and Sobbrio 2018; Schaub and Morisi 2020). In the United States, both structural county-level broadband access (Lelkes, Sood, and Iyengar 2017) and individual social media use (Bail et al. 2018; Allcott et al. 2020) have been found to increase partisan hostility and polarization. The findings also converge on the negative impact of digital media on institutional and political trust (Pietsch and Martin 2011; Im et al. 2014; Sabatini and Sarracino 2019; Zimmermann and Kohring 2020). While the trust-eroding effects of the internet and social media are welcomed when encouraging democratic development in authoritarian contexts, in democracies they risk undermining the legitimacy and effectiveness of political institutions.

In this study, I explore the impact of social media consumption on institutional trust in the European Union (EU) among its citizens. While the literature on the political consequences of internet penetration predominantly focuses on political competition at a national level, with few exceptions (De Wilde, Michailidou, and Trenz 2014; Baccini, Sudulich, and Wall 2016) the effects of social media consumption on attitudes toward the EU are underexplored. The boundary conditions of studies on national contexts are ill equipped to allow for generalizations to the EU, an international organization with a complex, supranational mandate. Different from national contexts, ordinary citizens are relatively less informed about and uninterested in EU politics, and their trust inclinations are therefore more susceptible to media effects (Brosius, van Elsas, and de Vreese 2019). Trust, however, is a critical component of diffuse support for EU policies, given that channels that democratic states typically derive their legitimacy from—including electoral accountability or citizen input and control in decision-making processes—are considerably weaker in the EU’s multilevel structure (Lord and Beetham 2001).

Empirically, using the Eurobarometer survey from 2019, I first show that higher social media use is associated with lower trust in the EU. More importantly, however, social media usage consumption exerts particularly detrimental effects in high-information environments. Specifically, in regions with wider and faster internet connections, those who more frequently use online social networks, tend to trust those networks, and receive information on EU affairs from them have less faith in the EU than those with similar consumption patterns who reside in regions with lower-quality internet access.

This study contributes to the literature on three grounds: First, it provides an EU-wide analysis on the effects of social media use on political trust. While some previous works have gathered evidence on the adverse effects of online exposure on EU attitudes using national samples (Baccini, Sudulich, and Wall 2016; Galpin and Trenz 2017), cross-national investigations that account for country-level heterogeneity and allow us to test the external validity of the hypothesized associations are limited and scattered (e.g., Ejaz 2020). Second, this study scrutinizes whether the effects of social media consumption are moderated by the quality of the information environment in which the user is embedded. By that, it provides a more nuanced view on how structural, macro-level factors interact with individual-level social media habits to shape political attitudes toward the EU.

Finally, third, the rapidly changing nature of social media platforms and user profiles and the gradual politicization of such channels necessitate regular updates of scholarly investigations on the subject, given that many of the earlier findings on the beneficial or null effects of online exposure for democracies have been contradicted by subsequent research (see Lorenz-Spreen et al. 2023). This study presents an up-to-date analysis on the year of the most recent European Parliament (EP) elections. Elections raise political debates and trigger the engagement and mobilization of voters and politicians on social media. The salience of European themes on online platforms increases, and social media users are exposed to a larger number of cues on the EU. Consequently, exploring the instrumental factors that influence political trust at a time when citizens were casting ballots to express their preferences on the EU helps us understand the immediate political repercussions of social media usage.

## Social Media and Institutional Trust

On a positive side, the decentralized nature of the internet and social media has a potentially liberating role. By helping break down monopolies over information enjoyed by states and political elites and disseminate it more democratically, web-based communication can boost voters’ political awareness, bolster critical perceptions of government performance, and ultimately make political institutions more accountable (Besley and Prat 2006). Social media serves as a medium through which people can raise their discontent and mobilize against existing political structures. By providing unobstructed communication channels for dissidents, it facilitates protests, not only in authoritarian countries, as crystallized during the Arab Spring, but also in democracies, as exemplified by the Occupy movement (Fergusson and Molina 2019). These effects not only diminish political trust in and allegiance to the political authority but can also foster democratic participation and higher levels of engagement (Brainard 2003).

Other effects of digital transformation are more troubling for democracies. Social media often turn into echo chambers (Sunstein 2017), in which individuals tend to consume media that reaffirm their preexisting dispositions and are posted by like-minded users. Algorithmic recommendations create “filter bubbles” that result in “mass audiences,” namely large groups consuming similar content, with minimal exposure to diverse perspectives and opinions (Flaxman, Goel, and Rao 2016). This triggers a vicious cycle of polarization, with individuals further entrenched in their positions at the expense of plurality and deliberation (Brundidge and Rice 2010).

Another disruptive matter concerns the content circulated on social media. It has been already well established that people are more inclined to react to negative news (Soroka and McAdams 2015), and this cognitive bias has given rise to a surge in negative advertising and campaigning in traditional media over the years (e.g., Geer 2012). This process has reached new heights with the advent of social media. Individuals have transformed from being passive consumers of negative news stories to active promoters by sharing them, commenting on them, or simply liking them. Worse, social media provides the means to spread false news. With editorial oversight and fact-checking virtually nonexistent, false news can spread faster and to a wider audience. For instance, during the 2016 US presidential elections, 6 percent of news posted on Twitter was reported as fake news (Grinberg et al. 2019). By fostering negativity and political cynicism, social media helps proliferate a narrative on the untrustworthiness of policymakers and contributes to declining political and institutional trust among its users.

Voters are relatively less informed and interested in European affairs, and online sources and social media have become a popular medium of keeping citizens informed about EU affairs (Vliegenthart et al. 2008). On the one hand, as Brosius, van Elsas, and de Vreese (2019) note, exposure to information on online platforms may increase citizens’ familiarity with the EU, encourage them to seek more accurate information on its capabilities and performance, and consequently increase trust.

On the other hand, heavier reliance on online sources will expose individuals to negative primes and misinformation (Mazzoleni 2014). On the supply side, in line with the populist narrative of building a direct connection to “real” people, an active engagement with social media has become an essential part of Eurosceptic parties’ political campaigns. While populist and Eurosceptical messages have a harder time being broadcasted on conventional media, which has traditionally been supportive of European integration, online platforms with no gatekeepers or filters are better able to reach their target audiences (Kriesi 2014). Consequently, social media has become a potent channel to politicize EU policies, with the aim of fostering Euroscepticism among voters (Bijsmans 2017; Usherwood and Wright 2017). This strategy also entails the use of fake and bot accounts that allude to a stronger support base and progressively expose real users to constant attacks against the EU and its values (Silva and Proksch 2021).

As a result of this politicization strategy, digital media posts on the EU are often dominated by a small number of posters who express strongly Eurosceptic views, whereas pro-European perspectives are often muted (Quinlan, Shephard, and Paterson 2015; Galpin and Trenz 2017). For instance, De Wilde, Michailidou, and Trenz (2014) found that during the EP election campaign of 2009, nearly two-thirds of reader comments posted in news platforms and blogs were either anti-European or diffusely Eurosceptic. In the subsequent 2014 EP election, the online discussion forums of news sites were once again predominantly populated by critical comments against both the EU and national institutions (Galpin and Trenz 2017). Moreover, social media platforms, particularly Twitter, played a stronger role in disseminating communication by political actors compared to 2009. Nulty et al. (2016) show that during the campaign period of the 2014 election, the majority of within-national hashtags on Twitter were from national parties with an anti-EU discourse. Similarly, during the Brexit campaigns, having analyzed 7.5 million tweets, Hänska and Bauchowitz (2017) found that Eurosceptic Twitter users outnumbered pro-European accounts and tweeted considerably more frequently.

Given the active presence of Eurosceptical actors on social media, coupled with an unequivocal share of negative and false stories and posts, it is intuitive to expect a strong association between social media use and attitudes toward the EU. Previous research lends credence to this expectation by demonstrating that using the internet as a source of political information makes citizens more critical of the EU (Baccini, Sudulich, and Wall 2016). Hence, *ceteris paribus*, those who use social media platforms more and gather news from social media are expected to be less trusting of the EU.

H1a: Those who use online social networks more frequently have less trust in the EU than those who use online social networks less frequently.

H1b: Those who receive information on EU affairs from online social networks have less trust in the EU than those who do not.

Methodologically, in the extant literature, data on online habits is predominantly acquired from surveys (Lorenz-Spreen et al. 2023, p. 91). However, self-reported use of the internet may be unreliable (Prior 2009). Moreover, there is a reverse causality problem (Schaub and Morisi 2020). Specifically, it is possible that those who trust the EU less, that is, Eurosceptics, use social media more to receive unfiltered news from their trusted politicians or to actively campaign for their positions on online platforms with lower entry barriers.

Critically, the volume of online media consumption is also a function of the quality of internet access. On the one hand, as the internet quality increases, both the time spent online and the volume of consumed digital content increase (Lelkes 2020). As users are provided with more choices in such high-information environments, the direct effects of the media environment are diluted, whereby individual dispositions and consumption patterns become more instrumental in shaping political attitudes (Prior 2007; Van Aelstet al. 2017).

On the other hand, a higher-bandwidth network connection enables users to send and receive more data at a time, resulting in substantially greater information consumption. In other words, users with similar levels of social media usage significantly vary in their consumption levels based on the quality of their connections. Given that the size of exposure to online content magnifies in line with the quality of access, theoretically, the adverse effects of social media usage on political trust should be particularly potent in regions with higher-quality internet.

H2a: Trust-eroding effects of social media use are stronger in regions with higher-quality internet than in regions with lower-quality internet.

H2b: Trust-eroding effects of receiving information on EU affairs from social media are stronger in regions with higher-quality internet than in regions with lower-quality internet.

Different forms of trust (i.e., generalized, social, institutional) are highly correlated to one another (Uslaner 2003), so we would assume that common underlying political psychological motivations drive trust in both political institutions (i.e., the EU) and social networks. Yet, the conditional effects of high-information environment stipulated in H2 should also apply to those who have greater trust in the credibility of the sources they consume from online social networks. Hence, the last hypothesis theorizes a further moderating effect between broadband coverage and trust in social media on EU attitudes.

H2c: Those who trust online social networks will have less trust in the EU in regions with higher-quality internet than in regions with lower-quality internet.

## Methods

The Eurobarometer survey 92.3 authorized by the European Commission fielded in November 14–December 13, 2019, constitutes the data source for the analysis (European Commission 2020). The mode of data collection consists of face-to-face interviews using stratified random sampling.^1^ The survey entails detailed questions on internet usage habits that permit empirical analysis on the hypothesized association between social media use and EU attitudes. Supplementary Material tables A1a and A1b report the question wordings, coding, and summary statistics of the study variables.

The dependent variable, trust in the EU, is gauged from the survey item that asks respondents whether they tend to trust the EU (For each of the following media and institutions, please tell me if you tend to trust it or tend not to trust it? 1 = tend to trust, 0 = tend not to trust).

The independent variable to test H1a is the self-reported usage frequency of social media (Could you tell me to what extent you *use* online social networks? *Use of Soc.Med.*), while to test H1b is whether respondents use online social networks to receive information about EU policies and institutions (When you are looking for information about the EU, its policies, its institutions, which of the following sources do you use? (multiple answers possible) *Info from Soc.Med.)* (1 = mentioned, 0 = not mentioned). Originally coded from 1 = no access to this medium to 7 = every day, *Use of Soc.Med* is standardized from 0 to 1 for easier comparison.

The independent variables to test H2a to H2c are interaction terms between social media usage habits and high-speed internet coverage. The first component of the interaction term stipulated in H2c is trust in social networks (*Trust in Soc.Med.)* (1 = tend to trust, 0 = tend not to trust). The second part of the proposed interactions is broadband availability at the regional, NUTS-2 level classified subdivisions within the EU countries.^2^ The corresponding data is extracted from Eurostat.^3^ For the analyzed sample, regional broadband availability varies moderately from a low of 70.8 percent to a high of 99.2 percent. Critics may argue, however, that in an advanced Web 2.0 era, most consumers access social media on their phones, rather than using broadband. Thus, as a robustness test, instead of broadband availability, I rerun the models using geocoded average mobile download speed data for the first three quarters of 2019 for each NUTS-2 region. The raw data is provided by Ookla, hosting Speedtest.net, a website through which users check the speed of their internet connections.^4^

I incorporate a battery of controls. Core demographic controls are age, gender, and education. To account for within-medium variation, consumption of info from other online channels, specifically, official websites, information websites, blogs, and video-hosting websites, are controlled for. The models account for cross-medium consumption by measuring the usage frequency of traditional media sources and whether the respondent receives information on the EU from such channels.^^5^^,^^6^^ Because both egotropic (Gabel and Palmer 1995) and sociotropic economic assessments affect EU attitudes (Anderson and Reichert 1995), two covariates are perceptions on national economy and the employment status. The logic of extrapolation proposes that citizens’ trust in the EU is closely related to their experiences in more visible, national institutions (Harteveld, van der Meer, and De Vries 2013); therefore, another control is trust in national governments. Finally, attachment to Europe is included to account for respondents’ self-identification with Europe and, hence, their general endorsement of the EU (Boomgaarden et al. 2011).

Unobserved macro-level factors may explain the structural variance in EU trust across regions (i.e., regional economic performance). Moreover, broadband availability and quality tend to be greater in regions with higher populations, GDP per capita, and favorable terrains. Hence, to account for structural differences and unobserved heterogeneity across regions, the model specifications use region-fixed effects. Although this model choice prevents us from estimating the direct effect of macro-level broadband availability on micro-level EU attitudes, it provides robust estimates to test the hypothesized cross-level interactions by eliminating omitted variable bias at the higher levels. Possible heteroscedasticity within clusters is addressed by robust standard errors. The models do not use survey weights.

## Results

Descriptively, 53 percent of Europeans express trust in the EU. With regard to social media habits, 47.4 percent of respondents state that they use social media every day, though 29.8 percent say they trust it. Only 15.7 percent receive information on the EU from social media, compared to 16.4 percent from official websites and 22.7 percent from information websites. In terms of traditional channels, TV and daily newspapers serve as the primary sources of information on EU affairs for 49.8 percent and 22.1 percent of respondents, respectively.

Moving away from aggregates, table 1 presents fixed-effects logistic estimates on EU trust to test the hypotheses. Model A reports on the main effects of social media usage. For each test of interaction effects subsequently, I first report the base model with only demographic controls and then the full specification.

**Table 1.** Social media use and EU trust: interaction effects with regional broadband access.

DV: EU TRUST (0-1)	(A)	(1)	(2)	(3)	(4)	(5)	(6)
Use of Soc.Med. (0-1)	−0.172	2.009	1.828		−0.203		−0.203
	**0.007**	**0.000**	**0.011**		**0.002**		**0.002**
Info from Soc.Med. (0-1)	−0.055		−0.025	3.027	1.893		−0.027
	**0.307**		**0.654**	**0.000**	**0.013**		**0.636**
Trust in Soc.Med. (0-1)	0.877		0.836		0.838	4.429	2.195
	**0.000**		**0.000**		**0.000**	**0.000**	**0.001**
Use of Soc.Med X Broadband		−0.023	−0.023				
		**0.000**	**0.005**				
Info from Soc.Med X Broadband				−0.034	−0.022		
				**0.000**	**0.012**		
Trust in Soc.Med X Broadband						−0.038	−0.016
						**0.000**	**0.028**
Use of TV (0-1)	0.554		0.534		0.535		0.531
	**0.000**		**0.000**		**0.000**		**0.000**
Use of newspaper (0-1)	0.108		0.079		0.080		0.081
	**0.128**		**0.282**		**0.276**		**0.268**
Info from TV (0-1)	0.042		0.038		0.035		0.036
	**0.319**		**0.394**		**0.426**		**0.414**
Info from newspaper (0-1)	0.226		0.265		0.265		0.265
	**0.000**		**0.000**		**0.000**		**0.000**
Demographic controls	YES	YES	YES	YES	YES	YES	YES
Attitudinal controls	YES	NO	YES	NO	YES	NO	YES
Region Fes	YES	YES	YES	YES	YES	YES	YES
Constant	−0.285	−0.516	−0.250	−0.376	−0.226	−1.012	−0.229
	**0.597**	**0.156**	**0.642**	**0.296**	**0.675**	**0.010**	**0.670**
Pseudo R²	0.2886	0.0759	0.2870	0.0709	0.2870	0.1143	0.2869
Observations	18,988	21,833	17,589	24,200	17,588	20,439	17,589

*Note:* Logit estimates with *p*-values in bold. Broadband is omitted, as it is fully collinear with NUTS-2 fixed effects. Full results are available in Supplementary Material section A2.

The results lend strong support for the proposed hypotheses, with the exception of H1b. Model A verifies that frequent users of social media have less trust in the EU, confirming H1a. In stark contrast, higher consumption of TV is significantly positively correlated to the outcome variable, whereas usage habits of newspapers and radio do not meaningfully affect EU trust.

Those who rely on social media to receive EU-related information are less trusting of the EU, though with a *p-*value of .307, this does not attain statistical significance. In contrast, those who receive EU-related information from two traditional sources—namely written press and the radio—as well as from two online channels—information websites and official websites—trust the EU significantly more (Supplementary Material table A2).

In the subsequent models, all the interaction terms of interest attain statistical significance, lending evidence for H2a–c. In regions with higher broadband access, each unit increase in social media use leads to lower trust in the EU. Similarly, in these regions, those who receive information about the EU from social media and trust social media become less trusting of the EU.

**Figure 1.**
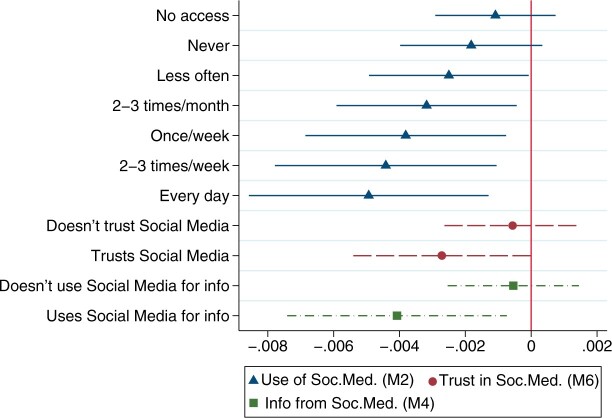
Average marginal effects of regional broadband coverage conditional on social media consumption habits (DV: EU Trust). Average marginal effects of regional broadband coverage for different values of *Use of Soc.Med*. (based on Model-2 estimates in table 1), *Info from Soc.Med.*(Model-4), and *Trust in Soc.Med.*(Model-6). The dots denote the median estimates, while the horizontal lines specify the 95 percent confidence intervals.

Figure 1 demonstrates the average marginal effects of *broadband* for values of social media usage. Confirming the theoretical expectations, each unit rise in the percentage of broadband coverage significantly decreases EU trust among those who use social media more than “less often,” receive information from it, and trust social media. Substantially, a person who uses social media every day residing in a region with 99 percent broadband coverage (e.g., Flevoland, Netherlands [NL23]) is 13.8 percent less likely to trust the EU than someone with the same usage but living in a region with 71 percent coverage (Limousin, France [FRI2], or Severozapaden, Bulgaria [BG31]).

**Figure 2.**
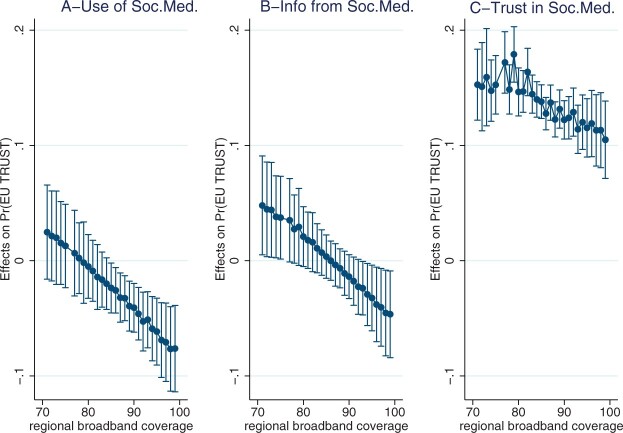
Average marginal effects of social media consumption habits conditional on regional broadband coverage (DV: EU Trust). Average marginal effects of *Use of Soc.Med.* (panel A), *Info from Soc.Med.* (panel B), and *Trust in Soc.Med*. (panel C) for observed values of regional broadband coverage. The dots denote the median estimates, while the horizontal lines specify the 95 percent confidence intervals.

To further demonstrate the difference in conditional effects in high- versus low-information environments, figure 2 plots the marginal effects of social media use across observed values of regional broadband access. The marginal effects of all three variables of interest follow a declining trajectory as regional broadband access rises. In regions with wider broadband access, unit changes in *Use of Soc.Med.* and *Info from Soc.Med.* exert a negative impact on trust levels, as proposed. The results in Panel B also show that in regions with lower broadband access, *Info from Soc.Med.* exerts significantly positive effects on trust levels. This finding points to a more nuanced, moderating impact of information environment: while at higher loads, receiving information from social media reduces political trust, at lower loads, it can instead boost support, arguably by enhancing political knowledge and cultivating interest (e.g., Allcott et al. 2020). A slightly different dynamic is observed for *Trust in Soc.Med*. (Panel C). Regardless of broadband access, those who trust social media also tend to trust the EU, arguably because some common underlying factors drive trust for both objects. Yet, there is a statistically significant difference between the marginal effects of *Trust in Soc.Med.* in low- versus high-information environments. In regions with greater broadband access, those who trust social media are significantly less likely to trust the EU compared to those in regions with low broadband access.

To determine the robustness of the findings, I run full models using geocoded mobile connection download speed data (*mobile speed*) at the regional level, replacing broadband access. The results in Supplementary Material table A4 largely verify the main findings. The interaction terms for both *Use of Soc.Med.* and *Trust in Soc.Med.* with *mobile speed* attain high statistical significance across models. The coefficients for the interaction term between *Info from Soc.Med.* and *mobile speed* do not attain statistical significance at the conventional levels with a *p*-value of .295, yet are in the hypothesized direction.

For an exploratory analysis, in Supplementary Material table A5, I also investigate the degree to which people differ in their media consumption patterns using principal component and latent class analyses on media usage and info source question sets. The results reveal a clear separation between those who exclusively consume traditional media and acquire political information from them, and those who almost entirely use online channels. This study demonstrated that while institutional trust remains high among this former group, for the latter, the so-called netizens, political trust is expected to remain highly fragile.

## Conclusion

This study showed that more frequent social media usage is associated with lower trust in the EU among European citizens, particularly so in regions with wider and faster internet infrastructures. Moreover, in such high-information environments, greater trust in social media sources and receiving information about the EU from digital channels exert particularly detrimental effects compared to regions with less efficient infrastructures. The evidence is based on cross-sectional Eurobarometer data, and it would therefore be erroneous to draw causal inferences. Yet, the results are suggestive of the detrimental impact of negative discourses and disinformation on social media platforms in eroding public support for European integration. A potential policy implication, therefore, points to a need for a concentrated effort to take concrete action against negative and false news about the EU being circulated on social media. Simultaneously, improving the visibility and engagement of EU institutions on online platforms and devising uninterrupted communication channels with European citizens would be warranted.

## Supplementary Material

nfad029_Supplementary_Data

## Data Availability

Replication data and documentation are available at https://doi.org/10.7910/DVN/ZLGOAO.

## Footnotes

1 Detailed information on the methodology of the Eurobarometer surveys in general can be accessed from the following link: https://www.gesis.org/en/eurobarometer-data-service/survey-series/standard-special-eb. Specific documentation about the survey used in the analysis is available at https://search.gesis.org/research_data/ZA7601.

2 According to the three-level NUTS (Nomenclature of Territorial Units for Statistics) classification, NUTS-2 regions refer to the second layer, corresponding to regions that often have their administrative structures and within the population threshold of a minimum of 800,000 and a maximum of 3 million inhabitants.

3 Broadband availability is measured by the percentage of households that are connectable (not necessarily connected) via a broadband technology. Data code TGS00048.

4 The data is publicly available at https://github.com/teamookla/ookla-open-data.

5 Both the usage frequency variables (standardized) and info sources variables vary between 0 and 1.

6 Usage frequency of internet is not controlled for, as it is expectedly highly correlated with social media consumption (alpha=0.72.6). However, the results are highly robust even if we include it as a covariate (corresponding tables in Supplementary Material section A3).

## References

[nfad029-B1] Allcott Hunt , Braghieri Luca, Eichmeyer Sarah, Gentzkow Matthew. 2020. “The Welfare Effects of Social Media.” American Economic Review 110:629–76.

[nfad029-B2] Anderson Christopher J. , Shawn Reichert M. 1995. “Economic Benefits and Support for Membership in the EU: A Cross-National Analysis.” Journal of Public Policy 15:231–49.

[nfad029-B3] Baccini Leonardo , Sudulich Laura, Wall Matthew. 2016. “Internet Effects in Times of Political Crisis: Online Newsgathering and Attitudes toward the European Union.” Public Opinion Quarterly 80:411–36. 27274571 10.1093/poq/nfv055 PMC4888570

[nfad029-B4] Bail Christopher A. , Argyle Lisa P., Brown Taylor W., Bumpus John P., Chen Haohan, Fallin Hunzaker M. B., Lee Jaemin, Mann Marcus, Merhout Friedolin, Volfovsky Alexander. 2018. “Exposure to Opposing Views on Social Media Can Increase Political Polarization.” Proceedings of the National Academy of Sciences of the United States of America 115:9216–21. 30154168 10.1073/pnas.1804840115 PMC6140520

[nfad029-B5] Besley Timothy , Prat Andrea. 2006. “Handcuffs for the Grabbing Hand? Media Capture and Government Accountability.” American Economic Review 96:720–36.

[nfad029-B6] Bijsmans Patrick. 2017. “EU Media Coverage in Times of Crisis: Euroscepticism Becoming Mainstream?” In Euroscepticism, Democracy and the Media, edited by Manuela Caiani and Simona Guerra, 73–94. UK: Palgrave Macmillan.

[nfad029-B7] Boomgaarden Hajo G. , Schuck Andreas R. T., Elenbaas Matthijs, De Vreese Claes H. 2011. “Mapping EU Attitudes: Conceptual and Empirical Dimensions of Euroscepticism and EU Support.” European Union Politics 12:241–66.

[nfad029-B8] Brainard Lori A. 2003. “Citizen Organizing in Cyberspace: Illustrations from Health Care and Implications for Public Administration.” American Review of Public Administration 33:384–406.

[nfad029-B9] Brosius Anna , van Elsas Erika J., de Vreese Claes H. 2019. “How Media Shape Political Trust: News Coverage of Immigration and Its Effects on Trust in the European Union.” European Union Politics 20:447–67.

[nfad029-B10] Brundidge Jennifer , Rice Ronald E. 2010. “Political Engagement Online: Do the Information Rich Get Richer and the Like-Minded More Similar.” In Routledge Handbook of Internet Politics, edited by Andrew Chadwick and Philip N. Howard, 144–156. New York: Routledge.

[nfad029-B12] Campante Filipe , Durante Ruben, Sobbrio Francesco. 2018. “Politics 2.0: The Multifaceted Effect of Broadband Internet on Political Participation.” Journal of the European Economic Association 16:1094–136.

[nfad029-B13] De Wilde Pieter , Michailidou Asimina, Trenz Hans-Jörg. 2014. “Converging on Euroscepticism: Online Polity Contestation during European Parliament Elections.” European Journal of Political Research 53:766–83.

[nfad029-B14] Ejaz Waqas. 2020. “Traditional and Online Media: Relationship between Media Preference, Credibility Perceptions, Predispositions, and European Identity.” Central European Journal of Communication 13:333–51.

[nfad029-B15] European Commission. 2020. Eurobarometer 92.3 (2019). GESIS Data Archive, Cologne. ZA7601 Data file Version 1.0.0, 10.4232/1.13564. Date accessed 1 January 2023.

[nfad029-B16] Falck Oliver , Gold Robert, Heblich Stephan. 2014. “E-Lections: Voting Behavior and the Internet.” American Economic Review 104:2238–65.

[nfad029-B17] Fergusson Leopoldo , Molina Carlos. 2019. “Facebook Causes Protests.” Documento CEDE, no. 41. https://repositorio.uniandes.edu.co/bitstream/handle/1992/41105/dcede2019-41.pdf?sequence=1&isAllowed=y. Date accessed 1 January 2023.

[nfad029-B18] Flaxman Seth , Goel Sharad, Rao Justin M. 2016. “Filter Bubbles, Echo Chambers, and Online News Consumption.” Public Opinion Quarterly 80:298–320.

[nfad029-B19] Gabel Matthew , Palmer Harvey D. 1995. “Understanding Variation in Public Support for European Integration.” European Journal of Political Research 27:3–19.

[nfad029-B20] Galpin Charlotte , Trenz Hans-Jörg. 2017. “The Spiral of Euroscepticism: Media Negativity, Framing and Opposition to the EU.” In Euroscepticism, Democracy and the Media: Communicating Europe, contesting Europe, edited by Manuela Caiani and Simona Guerra, 49–72. UK: Palgrave, Macmillan.

[nfad029-B21] Gavazza Alessandro , Nardotto Mattia, Valletti Tommaso. 2019. “Internet and Politics: Evidence from UK Local Elections and Local Government Policies.” Review of Economic Studies 86:2092–135.

[nfad029-B22] Geer John G. 2012. “The News Media and the Rise of Negativity in Presidential Campaigns.” PS: Political Science & Politics 45:422–27.

[nfad029-B23] Grinberg Nir , Joseph Kenneth, Friedland Lisa, Swire-Thompson Briony, Lazer David. 2019. “Fake News on Twitter during the 2016 US Presidential Election.” Science (New York, N.Y.) 363:374–78. 30679368 10.1126/science.aau2706

[nfad029-B24] Guriev Sergei , Melnikov Nikita, Zhuravskaya Ekaterina. 2021. “3g Internet and Confidence in Government.” Quarterly Journal of Economics 136:2533–613.

[nfad029-B25] Hänska Max , Bauchowitz Stefan. 2017. “Tweeting for Brexit: How Social Media Influenced the Referendum.” https://eprints.lse.ac.uk/84614/1/Hanska-Ahy__tweeting-for-brexit.pdf. Date accessed 1 January 2023.

[nfad029-B26] Harteveld Eelco , van der Meer Tom, De Vries Catherine E. 2013. “In Europe We Trust? Exploring Three Logics of Trust in the European Union.” European Union Politics 14:542–65.

[nfad029-B27] Im Tobin , Cho Wonhyuk, Porumbescu Greg, Park Jungho. 2014. “Internet, Trust in Government, and Citizen Compliance.” Journal of Public Administration Research and Theory 24:741–63.

[nfad029-B28] Kriesi Hanspeter. 2014. “The Populist Challenge.” West European Politics 37:361–78.

[nfad029-B2900] Lelkes Yphtach . 2020. “A Bigger Pie: The Effects of High-Speed Internet on Political Behavior.” Journal of Computer-Mediated Communication 25:199–216.

[nfad029-B29] Lelkes Yphtach , Sood Gaurav, Iyengar Shanto. 2017. “The Hostile Audience: The Effect of Access to Broadband Internet on Partisan Affect.” American Journal of Political Science 61:5–20.

[nfad029-B30] Lord Christopher , Beetham David. 2001. “Legitimizing the EU: Is There a ‘Post-Parliamentary Basis’ for Its Legitimation?” JCMS: Journal of Common Market Studies 39:443–62.

[nfad029-B31] Lorenz-Spreen Philipp , Oswald Lisa, Lewandowsky Stephan, Hertwig Ralph. 2023. “A systematic review of worldwide causal and correlational evidence on digital media and democracy.” Nature Human Behaviour 7:74–101. 10.1038/s41562-022-01460-1 PMC9883171 36344657

[nfad029-B32] Mazzoleni Gianpietro. 2014. “Mediatization and Political Populism.” In Mediatization of Politics: Understanding the transformation of western democracies, edited by Frank Esser and Jesper Strömbäck, 42–56. UK: Palgrave Macmillan.

[nfad029-B33] Nulty Paul , Theocharis Yannis, Popa Sebastian Adrian, Parnet Olivier, Benoit Kenneth. 2016. “Social Media and Political Communication in the 2014 Elections to the European Parliament.” Electoral Studies 44:429–44.

[nfad029-B34] Pietsch Juliet , Martin Aaron. 2011. “Media Use and Its Effect on Trust in Politicians, Parties and Democracy.” Australasian Parliamentary Review 26:131–41.

[nfad029-B3200] Prior Markus . 2007. Post-Broadcast Democracy: How Media Choice Increases Inequality in Political Involvement and Polarizes Elections. UK: Cambridge University Press.

[nfad029-B3500] Prior Markus . 2009. “Improving Media Effects Research through Better Measurement of News Exposure.” The Journal of Politics 71:893–908.

[nfad029-B35] Quinlan Stephen , Shephard Mark, Paterson Lindsay. 2015. “Online Discussion and the 2014 Scottish Independence Referendum: Flaming Keyboards or Forums for Deliberation?” Electoral Studies 38:192–205.

[nfad029-B36] Sabatini Fabio , Sarracino Francesco. 2019. “Online Social Networks and Trust.” Social Indicators Research 142:229–60.

[nfad029-B37] Schaub Max , Morisi Davide. 2020. “Voter Mobilisation in the Echo Chamber: Broadband Internet and the Rise of Populism in Europe.” European Journal of Political Research 59:752–73.

[nfad029-B38] Silva Bruno Castanho , Proksch Sven-Oliver. 2021. “Fake It ’Til You Make It: A Natural Experiment to Identify European Politicians’ Benefit from Twitter Bots.” American Political Science Review 115:316–22.

[nfad029-B39] Soroka Stuart , McAdams Stephen. 2015. “News, Politics, and Negativity.” Political Communication 32:1–22.

[nfad029-B40] Sudulich Laura , Wall Matthew, Baccini Leonardo. 2015. “Wired Voters: The Effects of Internet Use on Voters’ Electoral Uncertainty.” British Journal of Political Science 45:853–81.

[nfad029-B41] Sunstein Cass R. 2017. # Republic: Divided Democracy in the Age of Social Media. New Jersey: Princeton University Press.

[nfad029-B42] Usherwood Simon , Wright Katharine A. M. 2017. “Sticks and Stones: Comparing Twitter Campaigning Strategies in the European Union Referendum.” British Journal of Politics and International Relations 19:371–88.

[nfad029-B43] Uslaner Eric M. 2003. “Trust, Democracy and Governance: Can Government Policies Influence Generalized Trust?” In Generating Social Capital: Civil Society and Institutions in Comparative Perspective, edited by Marc Hooghe and Dietlind Stolle, 171–90. UK: Palgrave Macmillan.

[nfad029-B4300] Van Aelst, Peter, Jesper Strömbäck, Toril Aalberg, Frank Esser, Claes De Vreese, Jörg Matthes, David Hopmann, Susana Salgado, Nicolas Hubé, and Agnieszka Stępińska. 2017. “Political Communication in a High-Choice Media Environment: A Challenge for Democracy?” Annals of the International Communication Association 41:3–27.

[nfad029-B44] Vliegenthart Rens , Schuck Andreas R. T., Boomgaarden Hajo G., De Vreese Claes H. 2008. “News Coverage and Support for European Integration, 1990–2006.” International Journal of Public Opinion Research 20:415–39.

[nfad029-B45] Zimmermann Fabian , Kohring Matthias. 2020. “Mistrust, Disinforming News, and Vote Choice: A Panel Survey on the Origins and Consequences of Believing Disinformation in the 2017 German Parliamentary Election.” Political Communication 37:215–37.

