# Supplementary material for: Social Media Effects on Public Trust in the European Union
Source: Public Opin Q. 2023 Aug 3;87(3):749–63. doi: 10.1093/poq/nfad029 (PMC10662665; doi:10.1093/poq/nfad029)
Supplement: nfad029_Supplementary_Data [file nfad029_supplementary_data.pdf]

**Supplementary Material**

**Title Page**

# Social Media Effects on Public Trust in the European Union

Authors:

**Dr. OSMAN SABRI KIRATLI**

https://orcid.org/**0000-0001-9963-3542**

Associate Professor, International Trade Department

Bogazici University, Istanbul

& Visiting Research Fellow

WZB, Berlin

Email: osmansabrikiratli@boun.edu.tr, osmansabrikiratli@gmail.com

Tel: 0090 532 4763686

Address: Bogazici University, Hisar Kampus, Bebek 34342, Istanbul, Turkey

**Table of contents:**

**A1a- Summary Statistics**

**A1b- Question wordings (with the original question numbers)**

**A2- Social Media Use and EU Trust -Interaction Effects -Full Models**

**A3: Social Media Use and EU Trust -Interaction Effects with Regional Broadband Access- Use Internet included**

**A4- Social Media Use and EU Trust -Interaction Effects with Mobile Connection Speed**

**A5- An Exploratory Typology of Europeans' Media Consumption Habits**

**A1a- Summary Statistics**

| Variable | | Obs | Mean | Std. Dev. | Min | Max |
|---|---|---|---|---|---|---|
| **Individual Level** | | | | | | |
| EU TRUST | 0=do not trust 1=tend to trust | 28,384 | 0.53 | 0.50 | 0 | 1 |
| Use of Soc.Med. | Original from 1=every day to 7=no access to this medium (standardized between 0-1) | 26,733 | 0.66 | 0.38 | 0 | 1 |
| Trust in Soc.Med. | 0=do not trust 1=tend to trust | 25,882 | 0.30 | 0.46 | 0 | 1 |
| Info from Soc.Med. | 1=receive information on EU from social media | 32,511 | 0.16 | 0.36 | 0 | 1 |
| Gender | 1=man 2=woman | 31,564 | 1.54 | 0.50 | 1 | 2 |
| Age | Age exact | 31,564 | 51.04 | 17.76 | 18 | 99 |
| Education | 0=no formal education to 9=22 years of education | 30,997 | 5.70 | 2.66 | 0 | 9 |
| Unemployed | 0=no, 1=yes | 31,564 | 0.07 | 0.25 | 0 | 1 |
| Economic sit. (nation) | 1=very good to 4= very bad | 30,732 | 2.53 | 0.80 | 1 | 4 |
| Attachment Europe | 1=very satisfied to 4 not at all satisfied | 31,017 | 2.27 | 0.89 | 1 | 4 |
| Trust Nat.Gov. | 0=do not trust 1=tend to trust | 29,859 | 0.41 | 0.49 | 0 | 1 |
| Info from radio | 1=receive EU information from radio | 31,564 | 0.22 | 0.41 | 0 | 1 |
| Info from newspaper | 1=receive EU information from daily newspaper | 31,564 | 0.22 | 0.42 | 0 | 1 |
| Info from TV | 1=receive EU information from TV | 31,564 | 0.50 | 0.50 | 0 | 1 |
| Info official websites | 1=receive EU information from this source | 31,564 | 0.16 | 0.37 | 0 | 1 |
| Info information websites | 1=receive EU information from this source | 31,564 | 0.23 | 0.42 | 0 | 1 |
| Info from blogs | 1=receive EU information from this source | 31,564 | 0.04 | 0.20 | 0 | 1 |
| Info video hosting websites | 1=receive EU information from this source | 31,564 | 0.05 | 0.21 | 0 | 1 |
| Use of TV | Original from 1=every day to 7=no access to this medium (standardized between 0-1) | 26,923 | 0.94 | 0.17 | 0 | 1 |
| Use of radio | | 26,866 | 0.78 | 0.30 | 0 | 1 |
| Use of newspaper | | 26,837 | 0.63 | 0.33 | 0 | 1 |
| Use of Internet | | 26,783 | 0.79 | 0.34 | 0 | 1 |
| **Regional Level** | | | | | | |
| Broadband (%) | | 20,227 | 86.46 | 6.82 | 70.8 | 99.2 |
| Mobile Speed (download in mbps) | | 27,945 | 39.46 | 8.05 | 18.53 | 61.93 |

**A1b- Question wordings (with the original question numbers)**

**QA6a**-I would like to ask you a question about how much trust you have in certain media and institutions. For each of the following media and institutions, please tell me if you tend to trust it or tend not to trust it.

5- Online social networks

12- The (NATIONALITY) Government

14- The European Union

a- Tend to trust
b- Tend not to trust

**QD3**- Could you tell me to what extent you…?

1- Watch television on a TV set
2- Watch television via Internet
3- Listen to the radio
4- Read the written press
5- Internet
6- Use online social networks
a- Everyday/almost every day
b- Two or three times a week
c- About once a week
d- Two or three times a month
e- Less often
f- Never
g- No access to this medium

**QD5**- When you are looking for information about the EU, its policies, its institutions, which of the following sources do you use? (multiple answers possible)

a- Discussions with relatives, friends, colleagues
b- Daily newspapers
c- Television
d- Radio
e- Institutional and official websites (governmental websites, etc.)
f- Information websites (websites from newspapers, news magazines, etc.)
g- Online social networks

- h- Attending conferences, talks, meetings
- i- Other newspapers, magazines
- j- Blogs
- k- Video hosting websites
- l- Books, brochures, information leaflets
- m- Telephone (Info lines, Europe Direct, etc.)
- n- Other (SPONTANEOUS)

**QA1a**- How would you judge the current situation in each of the following?

2- The situation of the (NATIONALITY) economy

- a- Very good
- b- Rather good
- c- Rather bad
- d- Very bad

**QC1a**- Please tell me how attached you feel to…

4- Europe

- a- Very attached
- b- Fairly attached
- c- Not very attached
- **d-** Not at all attached

**A2- Social Media Use and EU Trust -Interaction Effects -Full Models**

| DV: EU TRUST | (A) | (1) | (2) | (3) | (4) | (5) | (6) |
|---|---|---|---|---|---|---|---|
| Age | -0.010 | -0.002 | -0.011 | -0.003 | -0.011 | -0.003 | -0.011 |
| | (0.001) | (0.001) | (0.001) | (0.001) | (0.001) | (0.001) | (0.001) |
| | **0.000** | **0.091** | **0.000** | **0.001** | **0.000** | **0.002** | **0.001** |
| Gender | 0.157 | 0.087 | 0.159 | 0.070 | 0.158 | 0.046 | 0.159 |
| | (0.037) | (0.029) | (0.038) | (0.027) | (0.039) | (0.031) | (0.038) |
| | **0.000** | **0.003** | **0.000** | **0.010** | **0.000** | **0.133** | **0.000** |
| Education | 0.052 | 0.097 | 0.053 | 0.084 | 0.053 | 0.102 | 0.053 |
| | (0.008) | (0.006) | (0.009) | (0.006) | (0.009) | (0.007) | (0.009) |
| | **0.000** | **0.000** | **0.000** | **0.000** | **0.000** | **0.000** | **0.000** |
| Use of Soc.Med. | -0.172 | 2.009 | 1.828 | | -0.203 | | -0.203 |
| | (0.064) | (0.515) | (0.719) | | (0.066) | | (0.066) |
| | **0.007** | **0.000** | **0.011** | | **0.002** | | **0.002** |
| Info from Soc.Med. | -0.055 | | -0.025 | 3.027 | 1.883 | | -0.027 |
| | (0.054) | | (0.057) | (0.525) | (0.756) | | (0.057) |
| | **0.307** | | **0.654** | **0.000** | **0.013** | | **0.636** |
| Trust in Soc.Med. | 0.877 | | 0.836 | | 0.838 | 4.429 | 2.195 |
| | (0.045) | | (0.047) | | (0.047) | (0.483) | (0.619) |
| | **0.000** | | **0.000** | | **0.000** | **0.000** | **0.000** |
| **Use of Soc.Med.X Broadband** | | -0.023 | -0.023 | | | | |
| | | (0.006) | (0.008) | | | | |
| | | **0.000** | **0.005** | | | | |
| **Info from Soc.Med. X Broadband** | | | | -0.034 | -0.022 | | |
| | | | | (0.006) | (0.009) | | |
| | | | | **0.000** | **0.012** | | |
| **Trust in Soc.Med. X Broadband** | | | | | | -0.038 | -0.016 |
| | | | | | | (0.006) | (0.007) |
| | | | | | | **0.000** | **0.028** |
| Trust-Gov. | 2.010 | | 2.046 | | 2.041 | | 2.042 |
| | (0.044) | | (0.046) | | (0.046) | | (0.046) |
| | **0.000** | | **0.000** | | **0.000** | | **0.000** |
| Attach-Europe | -0.652 | | -0.617 | | -0.618 | | -0.619 |
| | (0.025) | | (0.025) | | (0.025) | | (0.025) |
| | **0.000** | | **0.000** | | **0.000** | | **0.000** |
| Sit.-Nat.Econ | -0.198 | | -0.217 | | -0.218 | | -0.217 |
| | (0.030) | | (0.031) | | (0.031) | | (0.031) |
| | **0.000** | | **0.000** | | **0.000** | | **0.000** |
| Unemployed | -0.090 | | -0.072 | | -0.079 | | -0.075 |
| | (0.075) | | (0.078) | | (0.078) | | (0.078) |
| | **0.232** | | **0.359** | | **0.313** | | **0.340** |
| Use of TV | 0.554 | | 0.534 | | 0.535 | | 0.531 |
| | (0.123) | | (0.127) | | (0.127) | | (0.126) |
| | **0.000** | | **0.000** | | **0.000** | | **0.000** |
| Use of radio | 0.006 | | -0.020 | | -0.020 | | -0.019 |

| | | | | | | | |
|---|---|---|---|---|---|---|---|
| | (0.072) | | (0.075) | | (0.075) | | (0.074) |
| | **0.931** | | **0.787** | | **0.786** | | **0.800** |
| Use of newspaper | 0.108 | | 0.079 | | 0.080 | | 0.081 |
| | (0.071) | | (0.073) | | (0.073) | | (0.073) |
| | **0.128** | | **0.282** | | **0.276** | | **0.268** |
| Info from TV | 0.041 | | 0.038 | | 0.035 | | 0.036 |
| | (0.042) | | (0.044) | | (0.044) | | (0.044) |
| | **0.331** | | **0.394** | | **0.426** | | **0.414** |
| Info from radio | 0.127 | | 0.114 | | 0.114 | | 0.113 |
| | (0.051) | | (0.052) | | (0.052) | | (0.052) |
| | **0.012** | | **0.029** | | **0.029** | | **0.030** |
| Info from newspaper | 0.226 | | 0.265 | | 0.265 | | 0.265 |
| | (0.052) | | (0.053) | | (0.054) | | (0.053) |
| | **0.000** | | **0.000** | | **0.000** | | **0.000** |
| Info official websites | 0.091 | | 0.110 | | 0.105 | | 0.104 |
| | (0.050) | | (0.052) | | (0.052) | | (0.052) |
| | **0.071** | | **0.033** | | **0.042** | | **0.043** |
| Info information websites | 0.258 | | 0.255 | | 0.253 | | 0.252 |
| | (0.045) | | (0.046) | | (0.046) | | (0.046) |
| | **0.000** | | **0.000** | | **0.000** | | **0.000** |
| Info from blogs | -0.096 | | -0.091 | | -0.092 | | -0.085 |
| | (0.093) | | (0.101) | | (0.101) | | (0.101) |
| | **0.302** | | **0.368** | | **0.365** | | **0.400** |
| Info video hosting websites | -0.044 | | -0.006 | | -0.007 | | -0.006 |
| | (0.093) | | (0.098) | | (0.098) | | (0.098) |
| | **0.633** | | **0.951** | | **0.947** | | **0.953** |
| **Region FEs** | **YES** | **YES** | **YES** | **YES** | **YES** | **YES** | **YES** |
| Constant | -0.287 | -0.516 | -0.252 | -0.376 | -0.228 | -1.012 | -0.231 |
| | (0.540) | (0.366) | (0.538) | (0.360) | (0.539) | (0.394) | (0.538) |
| | **0.595** | **0.156** | **0.639** | **0.296** | **0.672** | **0.010** | **0.668** |
| Pseudo $R^2$ | **0.2886** | **0.0759** | **0.2870** | **0.0709** | **0.2870** | **0.1143** | **0.2869** |
| Observations | 18,988 | 21,833 | 17,589 | 24,200 | 17,588 | 20,439 | 17,589 |

Logit estimates with robust standard errors in parentheses and $p$-values in bold. Broadband is omitted as it is fully collinear with NUTS-fixed effects.

**A3: Social Media Use and EU Trust -Interaction Effects with Regional Broadband Access- Use of Internet included**

The models include *Use of Internet* as an additional control. Caution is warranted as *Use of Internet* is highly correlated with *Use of Soc.Med.* (0.726) and a VIF score of 26.82 indicates high multicollinearity (Model-A).

| DV: EU TRUST | (A) | (1) | (2) | (3) |
|---|---|---|---|---|
| Use of Soc.Med. | -0.293 | 1.466 | -0.281 | -0.283 |
| | (0.076) | (0.770) | (0.078) | (0.078) |
| | **0.000** | **0.057** | **0.000** | **0.000** |
| Info from Soc.Med. | -0.053 | -0.025 | 1.785 | -0.025 |
| | (0.054) | (0.057) | (0.758) | (0.057) |
| | **0.323** | **0.665** | **0.019** | **0.655** |
| Trust in Soc.Med. | 0.875 | 0.836 | 0.837 | 2.126 |
| | (0.045) | (0.047) | (0.047) | (0.620) |
| | **0.000** | **0.000** | **0.000** | **0.001** |
| **Use of Soc.Med.X Broadband** | | -0.020 | | |
| | | (0.009) | | |
| | | **0.022** | | |
| **Info from Soc.Med. X Broadband** | | | -0.021 | |
| | | | (0.009) | |
| | | | **0.017** | |
| **Trust in Soc.Med. X Broadband** | | | | -0.015 |
| | | | | (0.007) |
| | | | | **0.037** |
| Trust-Gov. | 2.014 | 2.047 | 2.044 | 2.044 |
| | (0.044) | (0.046) | (0.046) | (0.046) |
| | **0.000** | **0.000** | **0.000** | **0.000** |
| Attach-Europe | -0.648 | -0.616 | -0.616 | -0.617 |
| | (0.025) | (0.025) | (0.025) | (0.025) |
| | **0.000** | **0.000** | **0.000** | **0.000** |
| Sit.-Nat.Econ | -0.199 | -0.217 | -0.218 | -0.217 |
| | (0.030) | (0.031) | (0.031) | (0.031) |
| | **0.000** | **0.000** | **0.000** | **0.000** |
| Unemployed | -0.086 | -0.071 | -0.077 | -0.073 |
| | (0.075) | (0.078) | (0.078) | (0.078) |
| | **0.250** | **0.366** | **0.327** | **0.354** |
| Use of TV | 0.544 | 0.529 | 0.527 | 0.524 |
| | (0.123) | (0.127) | (0.127) | (0.126) |
| | **0.000** | **0.000** | **0.000** | **0.000** |
| Use of radio | -0.011 | -0.027 | -0.031 | -0.030 |
| | (0.072) | (0.075) | (0.075) | (0.075) |
| | **0.881** | **0.716** | **0.680** | **0.690** |
| Use of newspaper | 0.099 | 0.075 | 0.073 | 0.074 |
| | (0.071) | (0.073) | (0.073) | (0.073) |
| | **0.164** | **0.307** | **0.316** | **0.309** |
| Use of Internet | 0.294 | 0.142 | 0.196 | 0.202 |
| | (0.097) | (0.106) | (0.101) | (0.101) |
| | **0.003** | **0.178** | **0.051** | **0.045** |
| Info from TV | 0.040 | 0.037 | 0.035 | 0.036 |

| | | | | |
|---|---|---|---|---|
| | (0.042) | (0.044) | (0.044) | (0.044) |
| | **0.339** | **0.399** | **0.430** | **0.419** |
| Info from radio | 0.131 | 0.116 | 0.116 | 0.116 |
| | (0.051) | (0.052) | (0.052) | (0.052) |
| | **0.009** | **0.026** | **0.026** | **0.026** |
| Info from newspaper | 0.226 | 0.265 | 0.266 | 0.265 |
| | (0.052) | (0.053) | (0.054) | (0.053) |
| | **0.000** | **0.000** | **0.000** | **0.000** |
| Info official websites | 0.087 | 0.107 | 0.102 | 0.101 |
| | (0.050) | (0.052) | (0.052) | (0.052) |
| | **0.084** | **0.038** | **0.049** | **0.049** |
| Info information websites | 0.246 | 0.249 | 0.245 | 0.244 |
| | (0.045) | (0.047) | (0.047) | (0.047) |
| | **0.000** | **0.000** | **0.000** | **0.000** |
| Info from blogs | -0.098 | -0.090 | -0.092 | -0.085 |
| | (0.093) | (0.101) | (0.101) | (0.101) |
| | **0.288** | **0.370** | **0.365** | **0.397** |
| Info video hosting websites | -0.044 | -0.006 | -0.007 | -0.006 |
| | (0.093) | (0.098) | (0.098) | (0.098) |
| | **0.638** | **0.949** | **0.946** | **0.952** |
| **Demographic controls** | **YES** | **YES** | **YES** | **YES** |
| **Region FEs** | **YES** | **YES** | **YES** | **YES** |
| Constant | -0.432 | -0.323 | -0.328 | -0.334 |
| | (0.543) | (0.542) | (0.542) | (0.541) |
| | **0.427** | **0.552** | **0.545** | **0.538** |
| Pseudo $R^2$ | **0.2889** | **0.2871** | **0.2871** | **0.2870** |
| Observations | 18,987 | 17,588 | 17,588 | 17,588 |

Logit estimates with robust standard errors in parentheses and *p*-values in bold. Broadband is omitted as it is fully collinear with NUTS-fixed effects.

**A4- Social Media Use and EU Trust -Interaction Effects with Mobile Connection Speed**

| DV: EU TRUST | (1) | (2) | (3) |
|---|---|---|---|
| Use of Soc.Med. | 0.402 | -0.170 | -0.164 |
| | (0.246) | (0.064) | (0.064) |
| | **0.103** | **0.008** | **0.010** |
| Info from Soc.Med. | -0.058 | 0.196 | -0.056 |
| | (0.054) | (0.244) | (0.054) |
| | **0.285** | **0.422** | **0.296** |
| Trust in Soc.Med. | 0.878 | 0.878 | 1.786 |
| | (0.045) | (0.045) | (0.216) |
| | **0.000** | **0.000** | **0.000** |
| **Use of Soc.Med.X Mobile speed** | -0.014 | | |
| | (0.006) | | |
| | **0.017** | | |
| **Info from Soc.Med. X Mobile speed** | | -0.006 | |
| | | (0.006) | |
| | | **0.295** | |
| **Trust in Soc.Med. X Mobile speed** | | | -0.023 |
| | | | (0.005) |
| | | | **0.000** |
| Trust-Gov. | 2.012 | 2.011 | 2.005 |
| | (0.044) | (0.044) | (0.044) |
| | **0.000** | **0.000** | **0.000** |
| Attach-Europe | -0.650 | -0.651 | -0.653 |
| | (0.025) | (0.025) | (0.025) |
| | **0.000** | **0.000** | **0.000** |
| Sit.-Nat.Econ | -0.201 | -0.199 | -0.196 |
| | (0.030) | (0.030) | (0.030) |
| | **0.000** | **0.000** | **0.000** |
| Unemployed | -0.091 | -0.088 | -0.091 |
| | (0.075) | (0.075) | (0.075) |
| | **0.226** | **0.240** | **0.225** |
| Use of TV | 0.544 | 0.545 | 0.538 |
| | (0.123) | (0.123) | (0.123) |
| | **0.000** | **0.000** | **0.000** |
| Use of radio | 0.003 | 0.006 | -0.001 |
| | (0.072) | (0.072) | (0.072) |
| | **0.971** | **0.937** | **0.990** |
| Use of newspaper | 0.102 | 0.104 | 0.107 |
| | (0.071) | (0.071) | (0.071) |
| | **0.150** | **0.144** | **0.131** |
| Info from newspaper | 0.226 | 0.226 | 0.225 |
| | (0.052) | (0.052) | (0.052) |
| | **0.000** | **0.000** | **0.000** |
| Info from TV | 0.040 | 0.040 | 0.039 |
| | (0.042) | (0.042) | (0.042) |
| | **0.342** | **0.350** | **0.353** |
| Info from radio | 0.129 | 0.128 | 0.131 |
| | (0.051) | (0.051) | (0.051) |
| | **0.011** | **0.011** | **0.010** |
| Info official websites | 0.089 | 0.088 | 0.087 |

| | | | |
|---|---|---|---|
| | (0.050) | (0.050) | (0.050) |
| | **0.074** | **0.079** | **0.081** |
| Info information websites | 0.258 | 0.256 | 0.254 |
| | (0.045) | (0.045) | (0.045) |
| | **0.000** | **0.000** | **0.000** |
| Info from blogs | -0.097 | -0.096 | -0.096 |
| | (0.093) | (0.093) | (0.093) |
| | **0.295** | **0.301** | **0.299** |
| Info video hosting websites | -0.044 | -0.046 | -0.043 |
| | (0.093) | (0.093) | (0.093) |
| | **0.632** | **0.622** | **0.645** |
| **Demographic Controls** | **YES** | **YES** | **YES** |
| **Region FEs** | **YES** | **YES** | **YES** |
| Constant | -0.264 | -0.280 | -0.255 |
| | (0.540) | (0.540) | (0.538) |
| | **0.625** | **0.605** | **0.636** |
| Pseudo $R^2$ | **0.2886** | **0.2886** | **0.2892** |
| Observations | 18,966 | 18,966 | 18,966 |

Logit estimates with robust standard errors in parentheses and *p*-values in bold. Average Mobile Data Download Speed (mobile) is omitted as it is fully collinear with NUTS-fixed effects.

**A5- An Exploratory Typology of Europeans' Media Consumption Habits**

The results of this study established that social media consumption has an adverse effect on the EU trust, whereas higher usage of TV and gathering information from traditional sources, such as the written press and the radio, is associated with greater trust levels. This raises the question of the degree to which people differ in their consumption patterns. If citizens hold distinct and identifiable preferences with regards to online vs. traditional media and exclusively consume a particular medium, then we can better predict which groups would be more prone to hold favorable attitudes toward the EU and which would be more Euroskeptical.

The two questions on the usage frequency of media types and reliance on them as an information source allows us to explore underlying dimensions in respondent preferences and identify patterns. For this purpose, I conduct an exploratory factor analysis using principal component for factor extraction (PCF) on data for each question sets and an additional latent class analysis (LCA) for the multiple-response, info source variables. As the following table (A4a) reports, the factor analysis on media consumption data retains two factors with Eigenvalues over 1. The first factor is tapped by extremely high internet and social media use. Those who score high on factor1, who can be defined as netizens, use internet and social media heavily and are unlikely to watch TV. The second factor, in contrast, identifies a tight inter-relationship between three variables, each measuring the usage frequency of traditional media and almost nonexistent loadings for items gauging the usage of online channels. In other words, this analysis reveals clearly distinct consumption patterns between those who exclusively consume traditional media and those who almost entirely use online channels.

**Table A5a: Factor loadings on Media Consumption Data**

| **Item** | **Factor 1** | **Factor 2** |
|---|---|---|
| Use of TV | -0.167 | ***0.517*** |
| Use radio | 0.124 | ***0.769*** |
| Use of newspaper | 0.050 | ***0.754*** |
| Use internet | ***0.923*** | 0.093 |
| Use social media | ***0.922*** | -0.012 |

| | | |
|---|---|---|
| **Eigenvalue** | **1.746** | **1.435** |
| **Total variance explained %** | **34.9** | **29.7** |

Note: Extraction using Principal Components Analysis; orthogonal varimax rotation

This separation in usage frequencies closely shapes the preference for information source on EU affairs, Table A4b reports. Netizens who score high on Factor-2 tend to receive information from online blogs, video hosting sites, and social media and only occasionally use information websites. In contrast, those who are identified by Factor-1 rely heavily on three traditional media sources for acquiring political information. For this set of questions, the analysis retains an additional third dimension, which is defined by the items of *Info official websites* and *Info information websites*, largely capturing those who seek rather factual, more refined information from credible sources.

**Table A5b: Factor loadings on Info Source Data**

| **Item** | **Factor 1** | **Factor 2** | **Factor 3** |
|---|---|---|---|
| Info newspaper | ***0.694*** | -0.082 | 0.143 |
| Info TV | ***0.744*** | 0.053 | -0.137 |
| Info radio | ***0.762*** | 0.036 | 0.001 |
| Info official websites | -0.007 | 0.023 | ***0.826*** |
| Info information websites | -0.010 | 0.142 | ***0.773*** |
| Info social media | 0.035 | ***0.659*** | 0.133 |
| Info blogs | 0.005 | ***0.712*** | 0.077 |
| Info video hosting websites | -0.007 | ***0.685*** | 0.048 |
| **Eigenvalue** | **1.617** | **1.44** | **1.34** |
| **Total variance explained %** | **20.0** | **18.0** | **16.8** |

Note: Extraction using Principal Components Analysis; orthogonal varimax rotation

Though the previous allows us to ascertain the stimulating dimensions that lead respondents to answer questions on media consumption in particular patterns, for a more fine-grained categorization of latent groups with distinct consumption characteristics, I next run a latent class analysis on multiple-response info source questions. The latent class analysis based on generalized structural equation modeling extracts three groups, as Table-A-4c reports. The first group is tapped by a meagre 28.9% probability of relying on the TV for political information, accompanied by extremely low degrees of consumption of other mediums. This is the largest group, as 51.6% of the respondents are predicted to be in that class. The second group, with a 28.6% probability of class membership, consists of respondents who rely heavily on three traditional media sources—the TV (93%), radio (59.3%), and newspapers (50.7%)—to gather information on the EU. Finally, 19.9% of respondents are predicted to belong to Class-3. These respondents are more reliant on online channels and also utilize the TV to gather political news. Specifically, an average respondent in Class-3 is 66.1% likely to use information websites, 49.9% to use official websites, 39.8% to use social media, and 38.2% likely to turn to the TV for information.

**Table A5c: Latent Class Analysis Info Source Data**

| **Item** | **Class 1** | **Class 2** | **Class 3** |
|---|---|---|---|
| **Predicted Probability** | **51.6** | **28.6** | **19.9** |
| Info newspaper | 0.074 | **0.507** | 0.194 |
| Info TV | **0.298** | **0.930** | **0.382** |
| Info radio | 0.026 | **0.593** | 0.163 |
| Info official websites | 0.069 | 0.103 | **0.499** |
| Info information websites | 0.101 | 0.153 | **0.661** |
| Info social media | 0.083 | 0.123 | **0.398** |
| Info blogs | 0.008 | 0.018 | 0.174 |
| Info video hosting websites | 0.014 | 0.020 | 0.173 |

Note: Estimated means of each item on info source question set for each class. Latent class marginal means are extracted using generalized structural equation modeling.

In conjunction, these results suggest that two underlying dimensions shape European citizens' media consumption patterns and preferences. On the one end are the heavy consumers of online channels, with minimal engagement with traditional media. On the opposing end are consumers of traditional media who tend to largely discount online channels. The latent tendencies that stimulate consumption patterns are also reflected by class categorization based on preferences on receiving information on EU affairs. Leaving aside the apathetic citizens—alarmingly the largest group among respondents—those who rely on traditional media and those who utilize online channels for political information appear rather entrenched in their consumption habits. This study demonstrated that political trust remains high among this latter group, though for the former, the so-called netizens, institutional trust remains highly fragile.
